# A Human Antibody to the CD4 Binding Site of gp120 Capable of Highly Potent but Sporadic Cross Clade Neutralization of Primary HIV-1

**DOI:** 10.1371/journal.pone.0072054

**Published:** 2013-08-26

**Authors:** Johannes S. Gach, Heribert Quendler, Tommy Tong, Kristin M. Narayan, Sean X. Du, Robert G. Whalen, James M. Binley, Donald N. Forthal, Pascal Poignard, Michael B. Zwick

**Affiliations:** 1 Department of Immunology and Microbial Science, The Scripps Research Institute, La Jolla, California, United States of America; 2 Division of Infectious Diseases, University of California Irvine, Irvine, California, United States of America; 3 Torrey Pines Institute for Molecular Studies, San Diego, California, United States of America; 4 Altravax, Inc., Sunnyvale, California, United States of America; 5 IAVI Neutralizing Antibody Center, The Scripps Research Institute, La Jolla, California, United States of America; German Primate Center, Germany

## Abstract

Primary isolates of HIV-1 resist neutralization by most antibodies to the CD4 binding site (CD4bs) on gp120 due to occlusion of this site on the trimeric spike. We describe 1F7, a human CD4bs monoclonal antibody that was found to be exceptionally potent against the HIV-1 primary isolate JR-FL. However, 1F7 failed to neutralize a patient-matched primary isolate, JR-CSF even though the two isolates differ by <10% in gp120 at the protein level. In an HIV-1 cross clade panel (n = 157), 1F7 exhibited moderate breadth, but occasionally achieved considerable potency. In binding experiments using monomeric gp120s of select resistant isolates and domain-swap chimeras between JR-FL and JR-CSF, recognition by 1F7 was limited by sequence polymorphisms involving at least the C2 region of Env. Putative N-linked glycosylation site (PNGS) mutations, notably at position 197, allowed 1F7 to neutralize JR-CSF potently without improving binding to the cognate, monomeric gp120. In contrast, flow cytometry experiments using the same PNGS mutants revealed that 1F7 binding is enhanced on cognate trimeric Env. BN-PAGE mobility shift experiments revealed that 1F7 is sensitive to the diagnostic mutation D368R in the CD4 binding loop of gp120. Our data on 1F7 reinforce how exquisitely targeted CD4bs antibodies must be to achieve cross neutralization of two closely related primary isolates. High-resolution analyses of trimeric Env that show the orientation of glycans and polymorphic elements of the CD4bs that affect binding to antibodies like 1F7 are desirable to understand how to promote immunogenicity of more conserved elements of the CD4bs.

## Introduction

Despite more than two decades of innovative vaccine design efforts, numerous preclinical and clinical trials, as well as an improved molecular understanding of the envelope glycoprotein (Env) of HIV-1, a vaccine able to induce broadly neutralizing antibodies (bnAbs) to HIV-1 remains elusive [1], [2]. Neutralizing antibody (nAb) titers typically correlate with the protection conferred by many antiviral vaccines on the market today [3], and are widely expected to be important for protection against HIV-1 infection [4]–[11]. For HIV-1, the target of nAbs is a heavily glycosylated trimer of gp120 and gp41 heterodimers that is held together by non-covalent interactions [7], [10]–[14]. The gp120 subunit on Env trimers is responsible for sequential engagement initially with host cell receptor CD4, followed by binding to coreceptor (e.g. CXCR4 or CCR5) [15], as required to mediate fusion with and entry into host cells. Various mechanisms allow the virus to evade neutralization. These include: (i) a high mutation rate that creates an extraordinary sequence diversity of Env (http://www.hiv.lanl.gov/); (ii) epitope shielding by carbohydrates [16]; (iii) steric constraints that limit access to the recessed receptor binding sites [17]–[19]; and (iv) promotion of immunodominant but ineffective antibody responses in the host at least in part through production of non-functional forms of Env that may serve as decoys [20]–[22].

Despite difficulties in eliciting bnAbs to HIV-1 through vaccination, several bnAbs have been isolated from infected donors over the last two decades [23]. These include 2F5, 4E10, and 10E8 [24]–[29], directed to the membrane-proximal external region (MPER) of gp41 [9], [30]; 2G12 [31]–[33], directed to a conserved cluster of oligomannose glycans on the silent face of gp120 [34]; Monoclonal antibodies (mAbs) PG9 and PG16 [35], whose quaternary epitopes appear to be contained primarily within V2 of gp120 [36]; several recently described mAbs that bind to a conserved, glycan-dependent epitope cluster at the base of V3 [3], [37]; and bnAbs of the CD4 binding site (CD4bs) class. With respect to CD4bs bnAbs, b12 was the first to be described [38], [39], and targets a relatively rigid subsite in the CD4bs that includes the CD4 binding loop [40].

Recently, several additional CD4bs-directed bnAbs have been identified [8], [19], [41], [42]. Most notably, mAb VRC01 has been shown to bind to the CD4bs with a similar footprint and mode of recognition as the CD4 receptor itself [19], explaining at least in part its extraordinary breadth against over 90% of circulating HIV-1 isolates. These findings, and the surprising subsequent discovery of VRC01-like antibodies in different HIV-1 seropositive human donors have reinvigorated enthusiasm for the CD4bs as a vaccine target [8], [43], [44]. However, attempts to elicit broad nAbs against this epitope by vaccination have to date met with very limited success. Accessibility to the CD4 binding pocket represents an evolutionary tradeoff between sufficient exposure to allow receptor binding and protection from antibody recognition [17]. Thus, many CD4bs antibodies such as mAb b6 will bind tightly to monomeric gp120 but cannot bind to functional, trimeric Env spikes and cannot neutralize primary isolates [45]–[47]. This effect of ‘quaternary occlusion’ of the CD4bs is associated with spikes of primary isolates but not of T-cell line adapted strains of HIV-1 (e.g. MN) or other ‘tier 1’ isolates, where the CD4bs is more accessible [46], [48]–[50]. In order to recognize the recessed CD4bs on primary isolates, antibodies must contend with surrounding structures on the Env spike including glycans, V5 and the V1/V2 loop [18]. A solution to this problem appears to have been found with mAb VRC01 and some of its homologs [8], [43], [44], as well as with b12, although the breadth of neutralization by b12 is somewhat limited either by variations in sequence of the CD4 binding loop or by distal mutations that appear to affect accessibility to its epitope on the native Env trimer [51].

Here we describe 1F7, a human mAb isolated from immortalized peripheral blood lymphocytes from blood of HIV-1-positive volunteers. 1F7 has previously been reported to bind to gp120 [24] but its epitope and capacity for neutralization has hitherto not been described. We found that 1F7 neutralizes a modest range of generally resistant (‘tier 2’) primary isolates of various clades, at times with very high potency. Surprisingly however, some ‘tier 1’ viruses that are generally considered to be relatively neutralization-sensitive were found to be resistant to 1F7 neutralization, which we attribute to sequence polymorphisms that affect the binding site on monomeric gp120. Unlike other CD4bs antibodies such as b6, whose inability to neutralize primary HIV-1 is typically associated with quaternary occlusion of the CD4bs on trimeric spikes, 1F7 can potently neutralize a number of primary viruses but appears to use more polymorphic residues. In order to elicit CD4bs bnAbs like VRC01 but disfavor both b6-type and 1F7-type Abs, new approaches to immunization may be required.

## Materials and Methods

### Human mAbs and HIV-1 Env Mutants

MAbs 1F7, 2G12, 4E10, and 2F5 were obtained from Polymun Scientific, Vienna, Austria. D.R. Burton (TSRI, La Jolla, CA) kindly provided human mAbs b12 [52], b6 [46], and DEN3 [38], [53], [54]. VRC01 was a kind gift from J. Mascola (VRC, Bethesda, MD) [19]. MAbs 17b [55], 15e [56], and F425-B4e8 [57] were obtained from the IAVI NAC Reagent Repository after generous donations from J. Robinson (Tulane) and L. Cavacini (Beth Israel Deaconess Medical Center).

Domain-swap gp160 genes were generated using overlapping forward and reverse primers that bridge the JR-CSF-JR-FL boundary at each domain of interest. Full-length Env from JR-CSF (AY669726) and JR-FL (AY669728) in the pMAmp expression vector [58], [59] were used as templates for overlap-extension PCR that was carried out using Platinum Pfx polymerase (Invitrogen). Two or more PCR fragments containing a region of overlap were synthesized in a first step, and then the amplicons were assembled in a second step. PNGS mutant Env genes were generated using mutagenic primers (Integrated DNA Technologies) that change the targeted codons. Mutant DNA was ligated and transformed into *E. coli*. Following amplification and purification, plasmid DNAs were sequenced across the entire gp160 open reading frame to confirm the correct sequence.

### Neutralization Assay (TZM-bl Assay Format)

Pseudotyped virions were generated in HEK293T cells by co-transfection with different HIV-1 Env plasmids and the HIV-1 Env-deleted backbone plasmid pSG3ΔEnv (NIHARRRP; contributed by J. Kappes and X. Wu), as previously described [21]. Pseudotyped virus was added at a 1∶1 ratio to serially diluted mAbs (starting at 25 µg/mL) and incubated for 1 h at 37°C. TZM-bl reporter cells were then added (1∶1 by volume) at 1×10^4^ cells/well in a final concentration of 15 µg/mL DEAE-dextran. After a 48 h incubation at 37°C, the cells were washed, lysed, and finally developed with luciferase assay reagent according to the manufacturer’s instructions (Promega). Luminescence in relative light units (RLU) was measured using an Orion microplate luminometer (Berthold Detection Systems). All experiments were performed at least in duplicate. The extent of virus neutralization in the presence of antibody was determined at the 50% inhibitory concentration (IC_50_) in the absence of mAb.

### Neutralization Assay (Monogram Biosciences Assay Format)

Pseudotyped viruses capable of a single round of infection were produced as described elsewhere [60]. In brief, HEK293 cells were co-transfected with plasmids encoding Env libraries plus an HIV sub-genomic vector that contains a firefly luciferase indicator gene. After two days, recombinant pseudotyped viruses were harvested and incubated for 1 h at 37°C with serial four-fold dilutions of Ab. Subsequently, U87 cells that express CD4 plus the CCR5 and CXCR4 coreceptors were added to the virus/nAb mixture and incubated for three days. Virus infectivity was determined by measuring the amount of luciferase activity in U87 cell lysates. Recombinant viruses pseudotyped with Env from amphotropic murine leukemia virus were used to control for non-specific neutralization. Neutralizing activity is reported as the concentration of each mAb required to confer inhibition of infection (IC_50_) as follows:
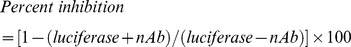



### Polyreactivity ELISA

Ninety-six well plates (Corning) were coated overnight (o/n) in 50**µl PBS at 4°C with 250 ng of antigen per well. Plates were washed with TPBS (phosphate buffered saline containing 0.05% Tween-20) and blocked with 4% non-fat dry milk (NFDM) in TPBS for 1 h at room temperature (RT). Meanwhile, dilution plates were prepared by serially diluting the human mAbs in 1% NFDM in TPBS. The mAbs were then transferred onto the blocked antigen-coated plate and incubated for 1 h at RT. After washing with TPBS, the plates were incubated for 1 h at RT with a horseradish peroxidase (HRP) labeled goat anti human Fab specific conjugate (Jackson ImmunoResearch Laboratories). Plates were finally washed with TPBS and developed using tetramethylbenzidine (TMB) substrate according to the manufacturer’s instructions (Pierce) and optical density (OD) at 450 nm was measured. To test for lipid reactivity, ELISA plates were coated with various lipid antigens (10 nM/well), diluted in methanol, and dried by evaporation. Plates were then blocked for 1 h at RT with 3% NFDM in PBS and washed with PBS. Serially diluted mAbs were added and incubated for 1 h at RT. Bound mAbs were probed as described above with the exception that PBS was used instead of TPBS.

### ELISA of Recombinant gp120

Ninety-six well plates were coated o/n at 4°C with 100 ng/well of purified monomeric gp120_JR-FL_ (Progenics Pharmaceuticals, Tarrytown, NY). Plates were washed with TPBS and blocked with 4% NFDM in TPBS for 1 h at RT. Serially diluted mAbs were then transferred onto the blocked antigen-coated plate and incubated for 1 h at RT. After washing with TPBS, plates were incubated for 1 h at RT with a HRP-labeled goat anti-human Fab-specific conjugate (Jackson ImmunoResearch Laboratories). MAb binding was detected as described above.

### ELISA of Env Captured from Detergent-solubilized Virions

Plates were coated with 100 ng/well of a gp120 Env specific anti-C5-antibody D7324 (Aalto), then washed and blocked, as above. After washing, HIV-1 pseudotyped virions solubilized in 1% Empigen (CALBIOCHEM, Cat. 324690) were added in the presence of 1% NFDM. In order to use a comparable amount of input gp120, we ran preliminary titrations of all lysates and normalized for 2G12 binding. Plates were incubated for 1–2 h at RT, and washed to remove unbound viral proteins. Serially diluted human mAbs were then added. MAb binding was detected as described above.

### Soluble CD4 Competition ELISA

Plates were coated with 100 ng/well of a gp120 Env specific anti-C5-antibody D7324, then washed and blocked as above. A constant concentration (1 µg/mL) of gp120 was added for 1 h at RT. Following washing; a fixed amount of sCD4 (20 µg/mL) mixed with a serial dilution of mAbs starting at a concentration of 2 µg/mL was added. The plates were incubated for 1 h at RT followed by a washing step to remove unbound proteins. MAb binding was detected using an anti-Fab HRP conjugate and plates were developed as above.

### Biotinylated mAb 1F7 Competition ELISA

Plates were coated with recombinant gp120_JR-FL_ (Progenics Pharmaceuticals, Tarrytown, NY) at a concentration of 1 µg/mL, then washed and blocked as above. After 1 h at RT, mAbs were serially diluted and mixed with a constant concentration of biotinylated 1F7 (0.5 µg/mL). After 1 h, plates were washed and bound mAb 1F7 was detected with a streptavidin HRP-conjugate (30 min at RT) and were developed as above.

### Native PAGE

BN-PAGE band shifts were used to analyze mAb binding to native, virion-derived Env trimers, as described previously [20], [61]–[63]. These assays used SOS-based parent and mutant pseudovirion VLPs [20], [61], [64]. The SOS mutant involves the substitution of cysteines at residues 501 and 605, which leads to the formation of a disulfide bond between gp120 and gp41. The SOS-based VLPs used were all truncated to remove most of the gp41 cytoplasmic tail, denoted gp160dCT, as described previously. This truncation increases the expression of native trimer, but has a negligible effect on the neutralization sensitivity of the pseudoviruses compared to those prepared using full length gp160 [62]. In BN-PAGE shifts, briefly, concentrated JR-FL SOS-VLPs were mixed with graded amounts of mAbs for 5 min. Particles were then washed with PBS and gently solubilized in 0.12% Triton X-100 in 1 mM EDTA/1.5 M aminocaproic acid and 1 µl of a protease inhibitor cocktail (Sigma). An equal volume of 2x sample buffer containing 100 mM morpholinepropanesulfonic acid (MOPS), 100 mM Tris-HCl, pH 7.7, 40% glycerol, and 0.1% Coomassie blue was then added. Samples were then loaded onto a 4–12% Bis-Tris NuPAGE gel (Invitrogen), using ferritin (Amersham) as a size standard. The gel was run at 4°C for 3 h at 100 V with 50 mM MOPS/50 mM Tris, pH 7.7, containing 0.002% Coomassie blue as cathode buffer and the same buffer without Coomassie blue as the anode buffer. The gel was then blotted onto polyvinylidene difluoride, destained, transferred to blocking buffer (4% NFDM in PBS) and probed with mAbs b12, 2G12, E51, 39F, 2F5, and 4E10, followed by an anti-human Fc alkaline phosphatase conjugate (Jackson ImmunoResearch). Trimer binding was measured via a depletion of the unliganded trimer using UN-SCAN-IT densitometry software (Silk Scientific) [63]. Experiments were run a minimum of three times to confirm the accuracy of IC_50_s. The molecular weights of trimer-nAb complexes were estimated relative to the estimated molecular weights of 420 and 140 kDa for JR-FL gp160ΔCT trimers and monomers, respectively [63].

### Flow Cytometry Analysis of mAb Binding to Cell Displayed Env

HEK 293T cells were transfected with envelope plasmid as described above, but omitting the pSG3ΔEnv backbone plasmid. Three days post transfection cells were harvested in FACS buffer (PBS, supplemented with 1% heat-inactivated fetal bovine serum, 1 mM EDTA and 25 mM HEPES, pH 7.0, and 0.2 µm filter sterilized) and aliquoted into a round-bottom 96-well plate at 2×10^5^ cells per well. After a centrifugation step, the cell pellet was resuspended in 50 µL of the appropriate mAb solution and incubated for 30 min at room temperature. Cells were then washed with FACS buffer and resuspended in R-phycoerythrin-conjugated goat anti-human IgG F(ab’)_2_-specific (Jackson ImmunoResearch). After another 30 min incubation step, cells were washed and finally transferred into FACS buffer containing SYTOX® Blue (Invitrogen). Doublets were excluded based on FSC and SSC, and the analysis based on cells that were negative for SYTOX® Blue. TreeStar FlowJo 7.6.4 was used for data evaluation.

## Results

### 1F7 Neutralizes Primary HIV-1 at Times with Extreme Potency but with Limited Breadth Across Clades

**Figure 1:**
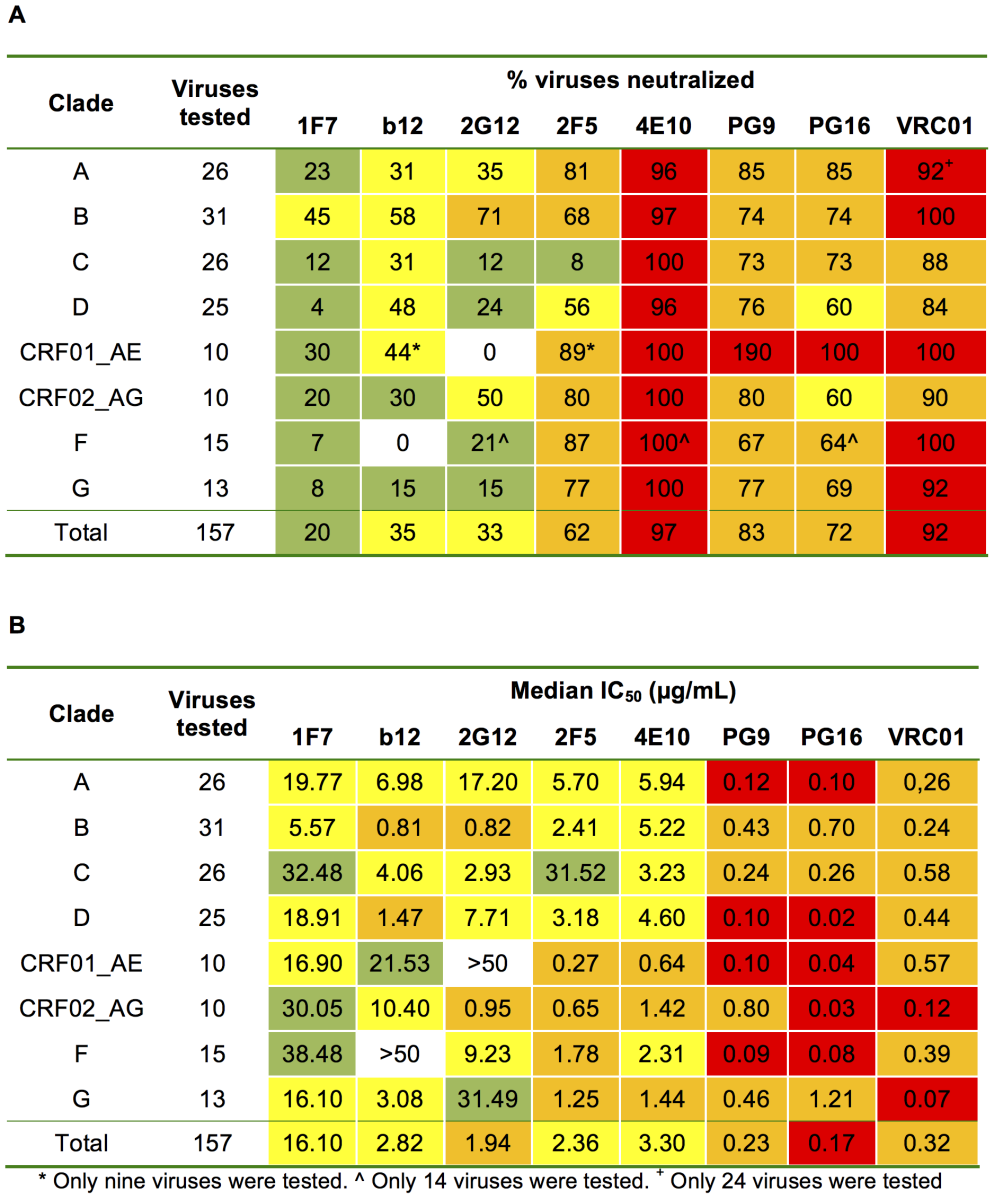
Neutralization activity of mAb 1F7 compared to mAb b12 and other broadly neutralizing HIV-1 antibodies. (A) The neutralization breadth is indicated by the percentage of neutralized viruses at an IC_50_<50 µg/mL. Boxes are color-coded as follows: white, no viruses neutralized; green, 1 to 30% of viruses neutralized; yellow, 31 to 60% of viruses neutralized; orange, 61 to 90% of viruses neutralized; red, 91 to 100% of viruses neutralized. The only tested clade AC virus is not included in the clade analysis, but is counted toward the total number of neutralized viruses. (B) The neutralization potency of the tested mAbs is shown as median IC_50_. Boxes are color-coded as follows: white, median potency >50 µg/mL; green, median potency between 20 and 50 µg/mL; yellow, median potency between 2 and 20 µg/mL; orange, median potency between 0.2 and 2 µg/mL; red, median potency <0.2 µg/mL. Color-coding as well as IC_50_ values for mAbs b12, 2G12, 2F5, 4E10, PG9, and PG16, which were obtained using the same assay, were taken from Walker and colleagues [35]. The neutralization potency of mAb 1F7 was measured in the Monogram Bioscience assay format.

MAb 1F7 was isolated from the immortalized B-cells of an HIV-infected donor [24], [65]. Details of its neutralizing activity, epitope, and sequence, however, have not been investigated. We initially discovered that this mAb could neutralize the relatively neutralization-resistant primary isolate JR-FL with extremely high potency (IC_50_≤0.1 µg/mL). We therefore examined the breadth of 1F7 neutralization (percent viruses neutralized at an IC_50_ below 50 µg/mL) against a comprehensive cross clade panel of 157 HIV-1 isolates (Figure S1). As summarized in Figure 1A, 1F7 neutralized 20% of HIV-1 isolates from all clades tested with an IC_50_<50 µg/mL. This degree of breadth is only slightly less than that of mAbs b12 (35%) and 2G12 (33%). In comparison, 2F5 (62%), PG16 (72%), PG9 (83%), VRC01 (92%), and 4E10 (98%) exhibited a more extensive neutralization breadth against the same panel [35], while in previous publications, CD4bs mAb HJ16 (36%) showed moderate breadth against a different multiclade panel of isolates [8], [41]. Similar to b12, 1F7 achieved its greatest neutralization breadth (45%) against clade B viruses including several ‘tier 2’ isolates. On the other hand, 1F7 was less potent than b12 against clades A, CRF01_AE, and CRF02_AG that were neutralized between 20% and 30% of the time. Only between 7% and 12% of C, F, and G clade viruses were neutralized by 1F7 (Figure 1A).

As indicated in Figure 1B, the median IC_50_ for mAb 1F7 was lowest for clade B viruses (5.57 µg/mL). A similar value was reached by 4E10 (5.22 µg/mL). In contrast, mAbs b12 (0.81 µg/mL), 2G12 (0.82 µg/mL), PG16 (0.70 µg/mL), PG9 (0.43 µg/mL), and 2F5 (2.41 µg/mL) revealed a considerably lower IC_50_, as reported previously [35]. The lowest median IC_50_ was observed with mAb VRC01 (0.24 µg/mL). Based on the total number of viruses tested (n = 157), 1F7 showed a median IC_50_ of 16.1 µg/mL, a much higher concentration compared to the other broadly neutralizing mAbs. Nevertheless, 1F7 potently neutralized (i.e. IC_50_≤1.34 µg/mL) clade A isolates 93UG077 and MGRM-A-009, as well as clade B isolates 92BR020, 93TH305, QH0692.42, SF162, NL4-3, and JR-FL, the latter three with IC_50_s ≤0.04 µg/mL (Figure S1).

### 1F7 Recognizes gp120 with Truncations of the V1/V2 and V3 Loops

**Figure 2:**
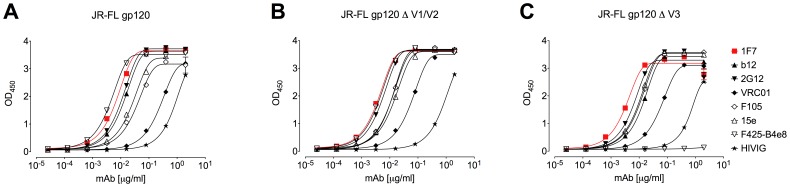
1F7 binding is independent of the gp120 variable loops V1/V2 and V3. Binding of 1F7 to recombinant gp120_JR-FL_ and its truncation mutants in a direct ELISA shows that 1F7 binding is independent of V1, V2, and V3. (A) 1F7 binding to wild type gp120_JR-FL_, (B) the variable loop truncated variants gp120_JR-FL_ ΔV1/V2, and (C) gp120_JR-FL_ ΔV3. Antibodies b12, VRC01, F105, 15e, 2G12, and HIVIG served as positive controls for each gp120, and F425-b4e8 to V3 is a negative control for the ΔV3 construct. Each curve is representative of at least three independent experiments performed in duplicate.

MAb 1F7 was confirmed to bind to soluble gp120 and not recombinant gp41, as previously reported [24], [65]. We then tested 1F7 ELISA binding to gp120_JR-FL_ as well as V1/V2- and V3-truncation mutants thereof, ΔV1/V2 and ΔV3 (Figure 2A, B, and C). 1F7 recognized all three gp120_JR-FL_ constructs approximately equally (EC_50_ ∼0.01 µg/mL). This was also true for control mAbs b12, 2G12, F105, VRC01, and 15e. In contrast, the V3-specific mAb F425-B4e8 failed to bind ΔV3 gp120_JR-FL_, as expected (Figure 2C). In a separate experiment, a V1/V2- and V3-dependent mAb, 4KG5 [66], bound to wildtype but not ΔV1/V2 gp120 (data not shown). The binding of mAb 1F7 is highly specific and not polyreactive, as 1F7 did not cross-react with lipid antigens such as cholesterol, cardiolipin, DOPE, or other auto antigens including dsDNA, histone, and transferrin at concentrations of up to 25 µg/mL (Figure S2). Thus, 1F7 binds specifically to an epitope on soluble Env (gp120) that is independent of V1/V2 and V3.

### 1F7 Competes with Soluble CD4 and CD4bs mAbs for Binding to gp120

**Figure 3:**
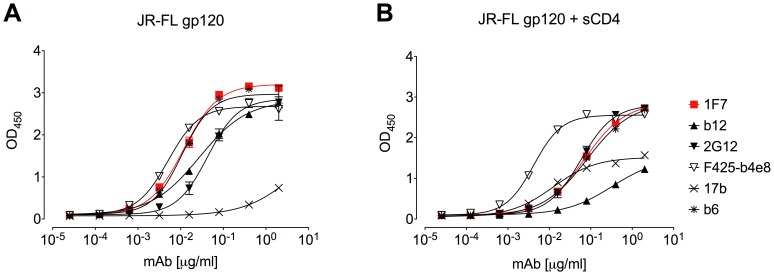
Soluble CD4 competes with mAb 1F7 for binding to gp120. MAb binding to gp120_JR-FL_ was measured using ELISA in the absence (A) or in the presence of sCD4 (B). MAb b12 was significantly inhibited by sCD4 (∼13-fold shift at half-maximum binding) whereas 1F7 exhibited a 6-fold shift and mAb b6 a 4-fold shift. The binding of control mAbs F425-B4e8 (V3) and 2G12 (binds to silent face of gp120) was not inhibited by sCD4, as expected. Each assay was performed in duplicate. Data are representative of at least 3 repeats.

**Figure 4:**
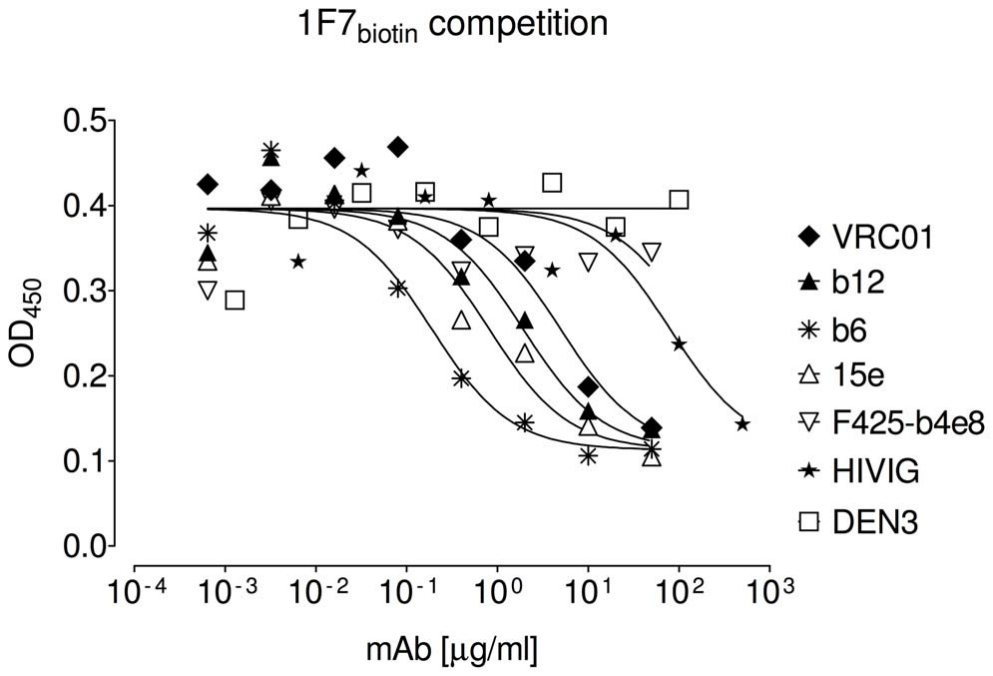
MAbs and HIVIG competition with 1F7 binding to gp120. MAbs b6, 15e, b12, HIVIG, F425-b4e8, VRC03, and VRC01 were co-incubated with a constant concentration of biotinylated mAb 1F7 and allowed to bind to immobilized gp120_JR-FL_. Curves were analyzed with GraphPad Prism 5.04 using a three-parameter fit with shared values for maximum and minimum binding. Data are representative of at least 3 titrations of each MAb against biotinylated mAb 1F7.

Given its relative indifference to V1/V2 and V3 as well as its capacity for cross-clade neutralization, we suspected 1F7 might target the CD4bs. We therefore examined the effect of soluble CD4 (sCD4) on 1F7 binding to gp120 by competition ELISA. Incremental concentrations of mAbs 1F7, b12, 2G12, b6, 17b, or F425-b4e8 were incubated in the presence or absence of excess sCD4 and incubated with HIV-1 gp120_JR-FL_ captured by an antibody directed to the gp120 C terminus (Figure 3A and B). The binding of control mAbs 2G12 and F425-B4e8 to gp120 was not inhibited by sCD4, as opposed to 17b whose binding was enhanced, as expected [12]. Only CD4bs mAbs b12, b6, and 1F7 were inhibited by sCD4 (Figure 3B). Next, we tested the ability of several mAbs and pooled immunoglobulin from HIV-1 infected donors (HIVIG) to compete with biotinylated mAb 1F7 for binding to immobilized gp120_JR-FL_. Here, CD4bs mAbs b6, 15e, b12, and VRC01, and to a lesser extent, HIVIG, interfered with 1F7 binding, but F425-B4e8 (V3), and DEN3 (non-HIV-1 control mAb) did not (Figure 4). These results are consistent with mAb 1F7 recognition of a CD4bs epitope.

### Gp120 Sequence Polymorphisms Affecting 1F7 Neutralization

**Figure 5:**
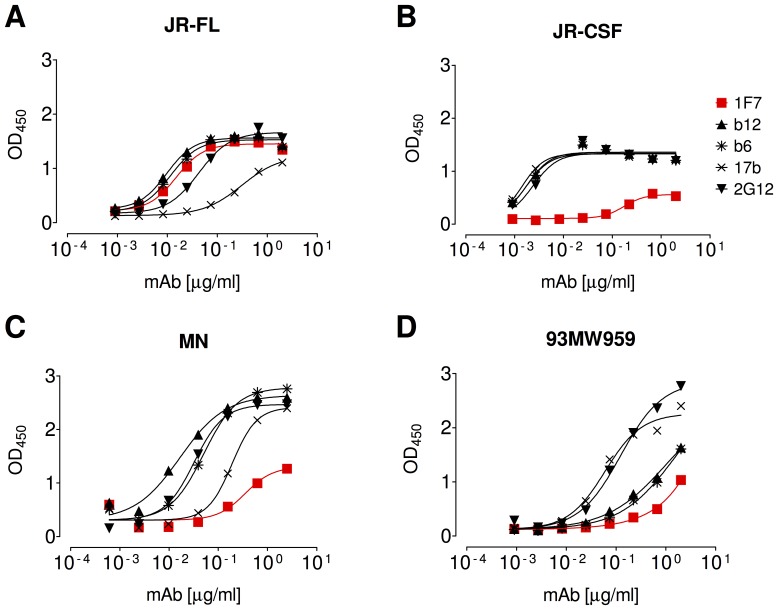
1F7 binding to sensitive and resistant Envs. Binding of 1F7 and other mAbs to detergent-solubilized Env from different isolates of pseudotyped HIV-1: (A) JR-FL, (B) JR-CSF, (C) MN, and (D) 93MW959. Env A (JR-FL) is 1F7-sensitive whereas Envs B-D are 1F7-resistant. Curves were analyzed with GraphPad Prism 5.04 using a four-parameter fit with shared values for minimum binding. Data are representative of at least 3 titrations of each MAb against the respective detergent-solubilized Env.

To understand the moderate neutralization breadth of mAb 1F7, we considered that neutralization by the CD4bs mAb b12 is regulated both by the primary sequence of CD4-contacting loops on gp120 and by quaternary constraints of adjacent protein surface and carbohydrate encircling the CD4 binding recess [67]. Both mechanisms might also operate here. One relevant observation is that 1F7 did not neutralize HIV-1 isolates MN and 93MW959 at the highest concentration tested (50 µg/mL), despite these being ‘tier 1’ viruses, against which multiple nAbs of different specificities are typically quite potent and in which the CD4bs is relatively exposed (Figure S1) [46], [48]–[50]. From these data, we can infer that quaternary masking may not explain the resistance to 1F7. Rather, sequence polymorphisms in the CD4bs may be responsible (e.g. gp120 JR-FL differs from MN, JR-CSF and 93MW959 by 97, 42, and 154 polymorphisms, respectively). To investigate this possibility, we performed ELISA binding studies in which Env from sensitive (JR-FL) and resistant (JR-CSF, MN, and 93MW959) isolates were captured from detergent-solubilized virions (Figure 5). Only Env from JR-FL and not other isolates showed strong reactivity with 1F7, which was comparable to that of b12 and 2G12 (Figure 5A–D). In contrast, and as a classic example of steric, quaternary occlusion [67], b6 bound tightly to gp120_JR-FL_ but did not neutralize this isolate, as expected. The binding epitopes of the capture antibody D7324 and the bnAb controls 2G12 and b12 are relatively conserved, particularly between JR-FL and JR-CSF. Thus, most likely one or more polymorphic residues in the resistant Envs disrupt the 1F7 epitope on both monomeric and native, trimeric gp120.

In a next step, we aligned the primary sequences of sensitive and resistant viruses from the neutralization analysis (Figure S3). Though many polymorphisms in regions relevant to CD4 recognition were present that might explain 1F7 neutralization [51], no consistent sequence patterns were evident among the panel viruses that clearly segregated with respect to 1F7 sensitivity and resistance (Figure S3).

### JR-CSF and JR-FL Env Chimeras Reveal that 1F7 Binding is C2-dependent, but that Neutralization is V1/V2-, C2-, and Glycan-dependent

**Figure 6:**
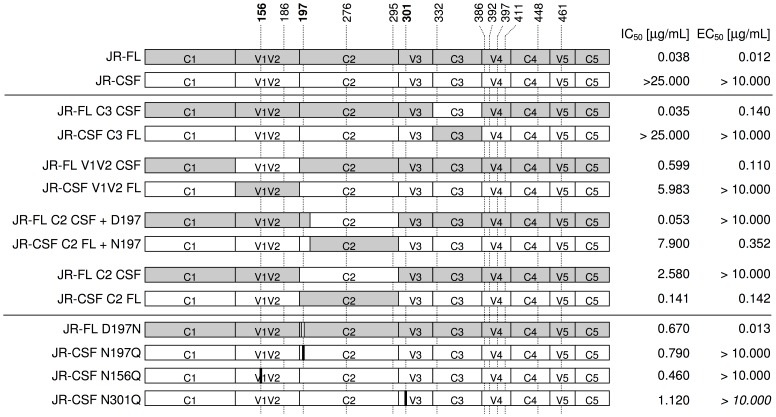
Neutralization potency (IC_50_) and binding to gp120 (EC_50_) JR-FL/JR-CSF chimeras as well as NGS mutants. Neutralization potency was assed using the TZM-bl assay format. The JR-FL sequence is represented in grey, JR-CSF in white, whereas residues that do not naturally occur in either are depicted black. Swapping of the V1V2 as well as the C2 domain caused major changes in sensitivity to 1F7. Of note, while a glycosylation at position 197 seems to render the virus more neutralization-sensitive, a swap of the more carboxy-terminal part of C2 was additionally required in order to impact antibody binding to detergent-solubilized gp120. EC_50_ data are representative of at least 3 titrations of each MAb against the respective JR-FL/JR-CSF chimeras.

**Figure 7:**
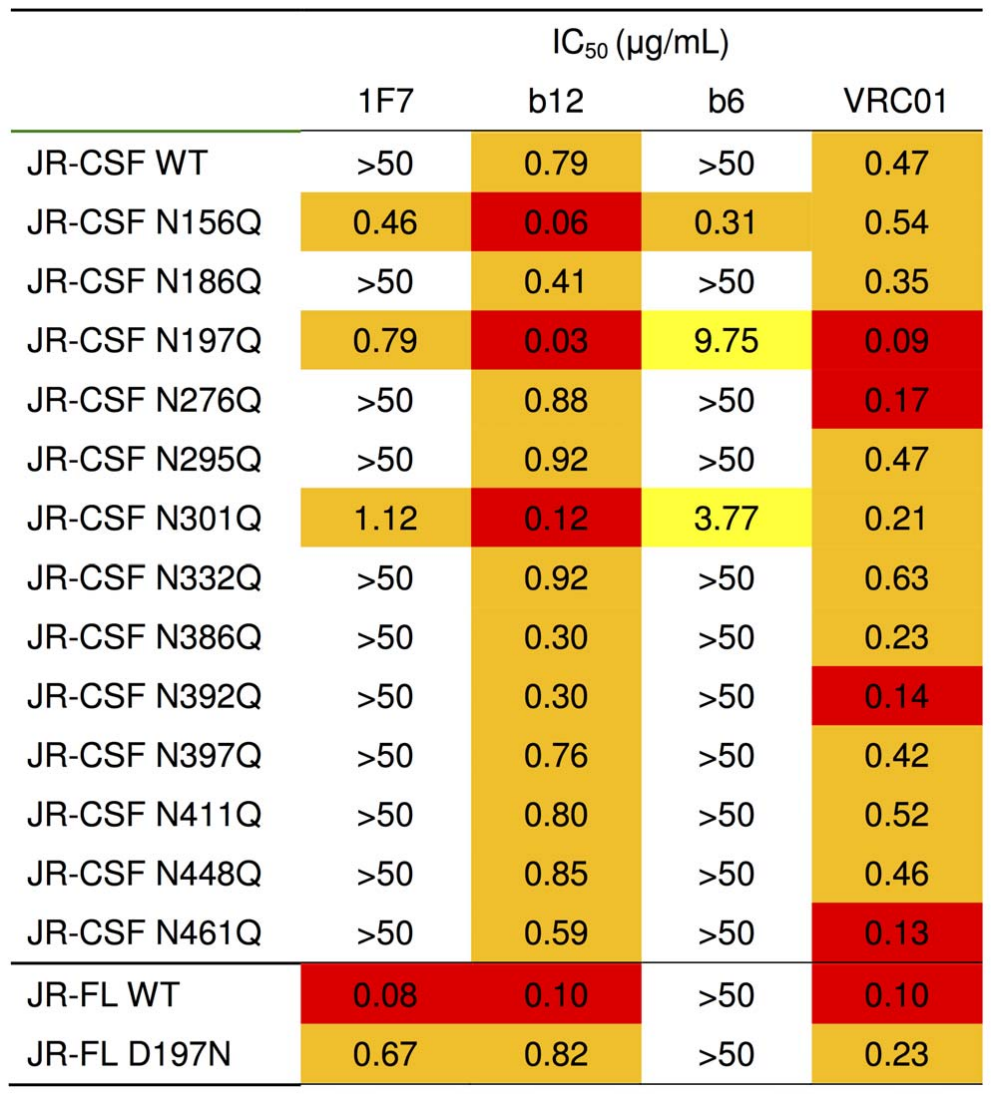
Neutralization (IC_50_s) of HIV-1 JR-CSF and JR-FL N-glycosylation mutants using 1F7 and other CD4bs mAbs. Neutralization assay was performed in the TZM-bl assay format. Boxes are color-coded as follows: white, median potency >50 µg/mL; green, median potency between 20 and 50 µg/mL; yellow, median potency between 2 and 20 µg/mL; orange, median potency between 0.2 and 2 µg/mL; red, median potency <0.2 µg/mL.

HIV-1 isolates JR-FL and JR-CSF represent two extremes of sensitivity and resistance to 1F7, respectively (Figure S1), even though they differ in only 42 out of 497 amino acids in gp120 and both are primary isolates from the same human host that are resistant to many other CD4bs mAbs like b6 [46], [48]. This afforded us an opportunity to use domain-swapped chimeras of the two viruses to delimit the 1F7 epitope using neutralization assays and by testing binding to detergent-solubilized gp120s. Several results with this chimerism analysis are notable. First, replacement of V1/V2 and C2 but not C3 regions of the two Envs greatly impacted both neutralization and binding by 1F7 (Figure 6). More specifically, introduction of the JR-FL-derived C2 (C2 FL) that includes the residue D197 from JR-FL into the JR-CSF background (JR-CSF C2 FL) restored 1F7 neutralization to near-wildtype JR-FL levels. Conversely, substituting JR-CSF C2 into a JR-FL background (JR-FL C2 CSF) decreased neutralization potency by more than 30-fold, while shifting the gp120-binding curve rightward to an EC_50_ above 10 µg/mL, which was the upper limit in our assay. Notably, the C2 region in JR-CSF contains the putative N-linked glycosylation site (PNGS) at position 197 that has previously been shown to modulate viral sensitivity to other CD4bs mAbs [51], [68]. In order to dissect the contributions of the PNGS at position 197 from the rest of the C2 region, chimeric viruses were tested that swapped C2 only while retaining the original amino acid at position 197 (Figure 6). As expected, mutants that contained D197 or Q197 were much more sensitive to 1F7 than their N197 equivalents (Figure 6, Figure 7).

### Glycans at gp120 Residues N156, N197 and N301 in gp120 Regulate JR-CSF Sensitivity to 1F7

As discussed above, one characteristic of JR-FL that may explain its sensitivity to 1F7 is a missing PNGS at position 197, which is extremely well conserved among other isolates, including JR-CSF. Together with certain other glycans on Env, this site has previously been shown to regulate mAb accessibility to the CD4bs [51]. To further test the effect of PNGS mutations, we used mAb 1F7 to probe a panel of JR-CSF pseudotyped viruses in which 13 individual PNGS were knocked out by mutation of the canonical motif, i.e. NXT/S to QXT/S. Of 13 JR-CSF PNGS mutants, three (N156Q, N197Q, and N301Q) were extremely sensitive to 1F7 (Figure 7). The typically non-neutralizing CD4bs mAb b6 also neutralized two of these variants (N197Q and N301Q), albeit less potently than 1F7. More subtle enhancements in neutralization were observed with b12 and VRC01, consistent with the idea that they already neutralize the parent virus effectively (Figure 7).

**Figure 8:**
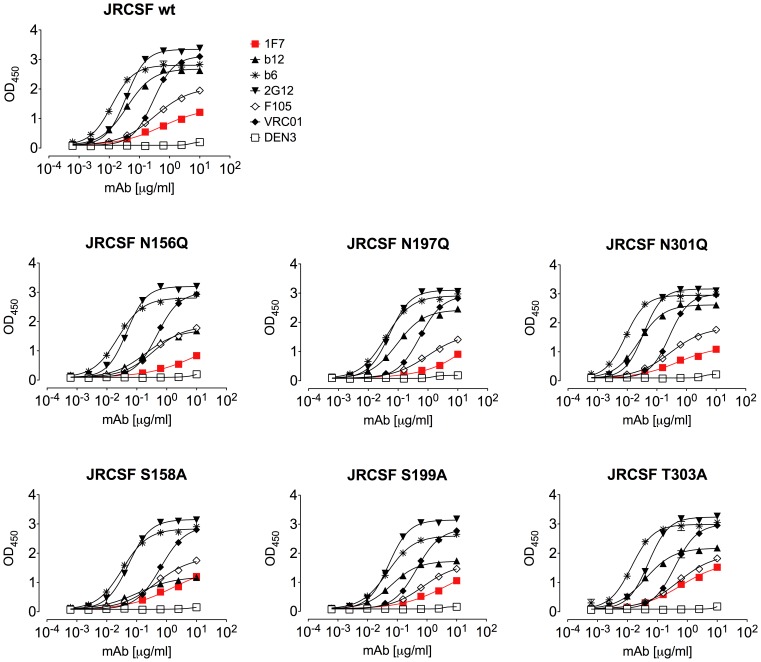
MAb 1F7 recognition of detergent-solubilized Env from neutralization-resistant wildtype JR-CSF and neutralization-sensitive glycovariants. 1F7 shows similar EC_50_s as well as relatively low maximum binding to gp120 relative to b12. 2G12 was used throughout the experiment to normalize the quantities of gp120. Each assay was performed in duplicate. Data are representative of at least 3 repeats.

**Figure 9:**
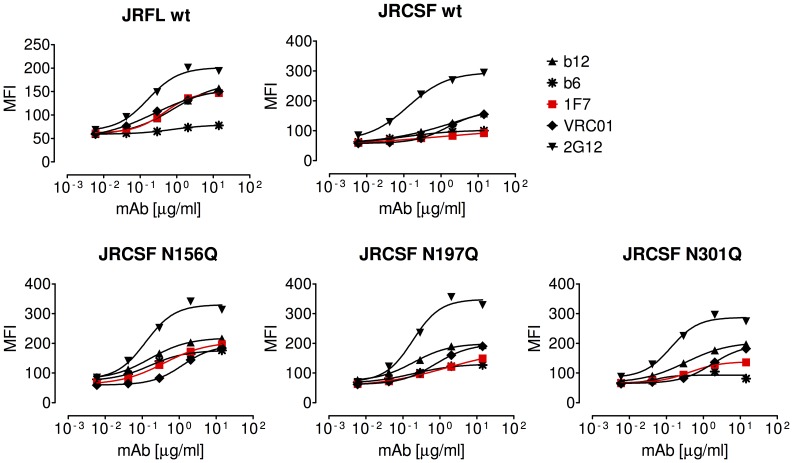
Flow cytometry analysis on cell surface expressed Env. 1F7 shows increased apparent binding to HIV-1 JR-CSF NGS mutant Env spikes displayed on cells as compared to wildtype spikes as measured using flow cytometry. Dashed lines represent the mean fluorescence (MFI) of control samples treated using secondary antibody only. Curves were prepared in GraphPad Prism 5.04 using a four-parameter fit. Data are representative of at least 3 titrations of each MAb against cell surface expressed Env.

We next tested whether the potent neutralization of 1F7 against the JR-CSF mutants N197Q, N156Q, and N301Q corresponded to a change in 1F7 binding to either soluble Env derived from virus lysates or to cell surface-expressed Env. As with wild-type JR-CSF, 1F7 only poorly bound to the mutants in ELISAs (Figure 8). In binding to cell surface-displayed Env, we observed slightly improved 1F7 binding to the more sensitive glycan mutants compared to the JR-CSF parent (Figure 9). Taken together, this suggests that the loss of glycans encircling the CD4bs better reveals a high affinity-binding site for 1F7 on trimeric Env but not on monomeric gp120.

The sensitivity of JR-CSF to 1F7 conferred by the N197Q glycan knockout mutation (Figure 7) opens up the possibility that adding a glycan at the unoccupied site (i.e. D197N) would render JR-FL more resistant to 1F7 neutralization. Indeed, the JR-FL D197N mutant was ∼8-fold more resistant to 1F7 and b12, and two-fold more resistant to VRC01 compared to wildtype JR-FL (Figure 7). This shows that introduction of a glycan at position 197 onto an Env background that naturally lacks this glycan can diminish access to the CD4bs. However, the effect is less marked than the effect of knocking out a naturally occurring glycan (PNGS) at this position. Taken together, our JR-CSF/JR-FL domain swap and JR-CSF PNGS mutant analyses indicate that elements located in the C2 region restrict 1F7 from binding JR-CSF monomeric gp120. In addition, V1/V2, C2, as well as glycans at positions 197, 156, and 301 cooperatively restrict antibody access to the CD4bs on JR-CSF native spikes.

### MAb and sCD4 Binding to Native Env Trimers

**Figure 10:**
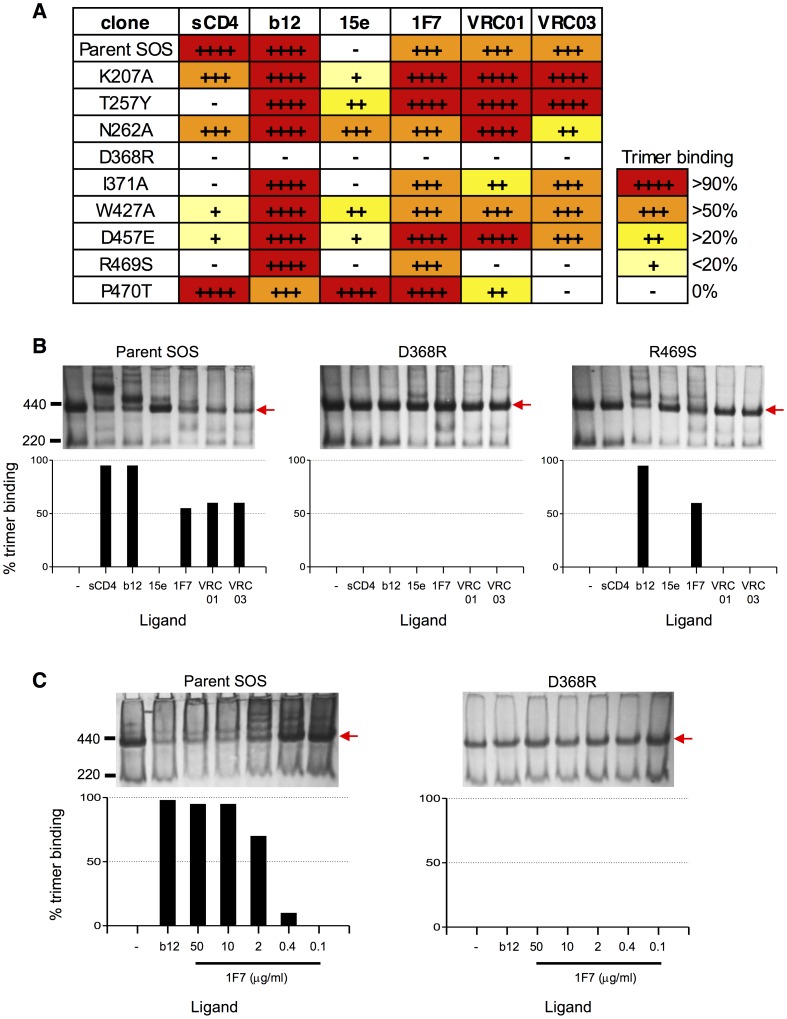
BN-PAGE analysis of mAb binding to point mutants of Env from HIV-1_JR-FL_. MAbs (30 µg/mL) were incubated with JR-FL SOS gp160ΔCT VLPs, which were then gently solubilized with non-ionic detergent and resolved using BN-PAGE. Western blots were then probed with a cocktail of anti-Env mAbs. MAb binding to unliganded Env trimers was measured as the relative depletion of the corresponding trimer band on the blot, as the trimers forms various mAb complexes. (A) Summary of effects of each point mutant on mAb binding. (B) Actual BN-PAGE Western blots showing mAb binding to parent, D368R and R469S SOS-VLPs. The molecular weights of ferritin standards are shown on the left and native Env trimers are indicated by an arrow to the right of each gel. A histogram beneath each gel quantifies the trimer-depleting effect of sCD4 and level of mAb binding. (C) Titration of the 1F7 mAb against parent and D368R SOS-VLPs. Histograms are used as in Panel B.

Prior studies have shown that particular mutations in Env can have differential effects on b12 binding, depending on the context of the mutation in either monomeric gp120 or native Env trimers [67]. Therefore, we assessed 1F7 binding to native JR-FL trimers using mutants selected based on a prior report of JR-CSF Env mutations that affect the CD4bs [67]. As many of these mutants adversely affect CD4 binding and thus make infectivity/neutralization assays impossible, we visualized mAb-trimer binding using BN-PAGE shift assays [69]. We observed that the parent Env JR-FL trimers were efficiently recognized by sCD4, b12, 1F7, VRC01, and VRC03, but not by the typically non-neutralizing CD4bs mAb 15e (Figure 10A and B). Correspondingly, all of these ligands except 15e also effectively neutralize the JR-FL isolate.

Out of the nine substitutions in our JR-FL Env trimer panel, six adversely affected sCD4 binding in the BN-PAGE assay (Figure 10A). Notably, although K207A, N262A, and P470T were previously shown to ablate sCD4 binding to monomeric JR-CSF gp120 [67], they had little or no effect on sCD4 binding to JR-FL native trimers (Figure 10A)**.** Only the D368R mutant knocked out binding of all the neutralizing ligands in our panel, including 1F7. Some mutants promoted 15e binding to trimers. This was most notable for N262A and P470T, and suggests a more “relaxed” quaternary structure that grants access to a CD4bs mAb that cannot otherwise recognize parental Env trimers.

Overall, more mutants affected recognition of Env trimers by sCD4 than was the case with any of the mAbs (namely mutants T257Y, D368R, I371A, W427A, D457E, and R469S). MAb 1F7 had a similar profile to that of b12 in being affected only by D368R (Figure 10A and B), whereas mAb VRC01 and its somatic variant VRC03 were sensitive to mutation R469S of gp120 in the C5 region (Figure 10A and B). Additionally, VRC03 was uniquely sensitive to P470T. To quantify the effect of D368R mutation, we titrated mAb 1F7 against parent JR-FL and D368R mutant trimers (Figure 10C). We found that 1F7 saturated the parent trimer at 2 µg/mL. In contrast, 1F7 binding to the D368R mutant was completely ablated, even at the highest concentration tested (50 µg/mL). Overall, the BN-PAGE results confirm that 1F7 binds to a D368R-sensitive epitope overlapping the CD4bs on trimeric Env, and that, with this mutant Env trimer panel at least, 1F7 has a binding profile that is more akin to that of b12 than the other CD4bs mAbs tested here.

### IgG 1F7 Derives from a Different Germline than Other CD4bs bnAbs

**Figure 11:**
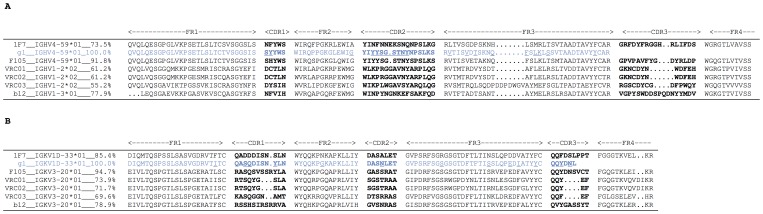
1F7 sequence alignment. Amino acid alignment of the 1F7 (A) heavy chain and (B) light chain variable regions along with several other CD4bs antibodies. The 1F7 germline (gl) is shown in blue and residues that differ between 1F7 and its germline sequence are underlined. (A) Antibody F105 and 1F7 share the same variable heavy chain germline family IGHV4-59*01. However, the 1F7 heavy chain sequence is less conserved than the one of F105 (73.5% versus 91.8%). (B) The variable light chain of 1F7 derives from a different germline gene (IGKV1D-33*01) than the other CD4bs antibody light chain fragments.

To determine whether 1F7 shares a common progenitor with other broadly neutralizing CD4bs reported previously, we aligned the variable IgG heavy and light chain amino acid sequences of 1F7, VRC01, VRC02, VRC03, b12, and F105 (Figure 11). Heavy chain alignments are shown in Figure 11A. As reported previously, mAbs VRC01, VRC02, and VRC03 share the same germline VH gene IGHV2*02 with a 61.2%, 61.2%, and 55.2% homology to germline at the amino acid level, respectively [8]. The heavy chain of b12 derives from germline gene IGHV1-3*01 with a homology to germline of 77.9%. Interestingly, 1F7 and the weakly neutralizing CD4bs mAb F105 belong to the same germline family IGHV4-59*01. However, F105 shares a higher homology to the germline gene IGHV4-59*01 (91.8%) than 1F7 (73.5%). F105 and 1F7 share sequence homology of only 65%, which is suggestive of distinct lineages that diverged relatively early on in their respective affinity maturation pathways. Considering IgG light chains, VRC01, VRC02, VRC03, b12, and F105 were all derived from the same germline gene IGKV3-20*01 (Figure 11B). F105 and b12 showed highest homology to the germline (94.7% and 78.9%, respectively) followed by VRC01 (73.9%), VRC02 (71.7%), and VRC03 (69.6%). In contrast, the light chain of mAb 1F7 is most closely matched to the IGKV1D-33 germline gene with a homology of 85.4%.

## Discussion

MAb b12 was the first reported broadly neutralizing mAb against HIV-1 and identified the CD4bs as a prime target for vaccine design [52]. The recent identification of the highly potent and broad-spectrum VRC01 mAb and subsequent discovery of VRC01 homologs in different individuals has boosted this enthusiasm [19], [43], [44]. However, vaccine approaches so far have failed to elicit nAbs of similar potency and breadth [6], [70], [71]. Most reported mAbs that fall into the CD4bs competition class, for example b6, b13, F105, F91, and 15e, exhibit very limited potency and/or breadth against primary isolates [18]. 1F7 belongs to a new, intermediate category, exhibiting neutralization breadth that is inferior to that of VRC01 and b12, but superior to the more typical non-neutralizing CD4bs mAbs like b6, 15e, or F105, and occasionally capable of extreme potency. Another recently described CD4bs mAb, HJ16, also appears to belong to this intermediate category. HJ16 has been shown to exhibit moderate breadth, but extraordinary potency against certain ‘tier 2’ isolates although with a neutralization profile that is distinct from that of 1F7 [41]. However, HJ16 is an unconventional CD4bs mAb, as it is not dependent on the D368 residue that commonly disrupts recognition by CD4bs Abs including 1F7, and the HJ16 epitope involves amino acids that are typically targeted by CD4-induced mAbs.

BN-PAGE band-shift analysis using soluble Env trimers derived from infectious pseudoviruses indicated a common dependence on D368 for all five CD4bs Abs tested herein. The very broadly neutralizing mAbs, VRC01 and VRC03, were, besides the D368 mutation, uniquely dependent on residues of the V5 loop (e.g. R469). This dependence on V5 is consistent with the proximity of V5 to the heavy chain/light chain interface of VRC01 bound to gp120 [19]. Thus, 1F7 appears to have more in common with b12 in terms of lesser neutralization breadth and lack of sensitivity to V5 mutations. A distinct angle of approach and positioning of antibody is crucial to effective CD4bs recognition, considering that the Fab arm of an antibody is approximately double the width of CD4. Hence, successful targeting of nAbs to this recessed site appears to be associated with narrow avoidance of steric blocks from V1/V2, V5, and proximal glycans, as well as a select choice of conserved residues within the CD4bs. These features appear to be largely fulfilled by mAb VRC01, which also does not require conformational changes in the envelope spike to reach its recessed target [72]. Although the epitope of 1F7 had not been described until the present study, it is notable that 1F7 was recently shown to induce shedding of gp120 from spikes of primary HIV-1; b12, but not VRC01 also mediates this shedding effect [22], [72], [73]. Here again, 1F7 shares a functional property with b12 that VRC01 lacks, perhaps again attributable in part to a requirement of conformational change on binding to spikes with b12 and 1F7 but not with VRC01.

The neutralization profile of 1F7 (Figure S1) exhibits several points of note. First, 1F7 neutralized two tier 2 isolates, PVO and TRO, which were resistant to b12. Second, 1F7 was unable to neutralize the highly sensitive tier 1 isolates MN or 93MW959, even though the Env spikes of these isolates are thought to have a particularly open and/or labile conformation [74]. In ELISA and flow cytometry binding experiments, this was explained by inefficient recognition of the CD4bs of these viruses both on detergent-solubilized (monomeric) and native (trimeric) gp120. Likewise, 1F7 was unable to neutralize JR-CSF or recognize its Env from detergent-lysed virus, even though this isolate shares high sequence homology with the isolate JR-FL, the latter of which is exceptionally sensitive to 1F7 neutralization (Figure S1). Many studies using certain non-neutralizing mAbs to the CD4bs like b6, F105, 15e, and other murine mAbs have documented a ‘quaternary occlusion’ effect, in which the mAbs bind tightly to conserved epitopes overlapping the CD4bs on monomeric gp120 but cannot neutralize and do not recognize functional, trimeric Env [45], [46], [51], [67]. Several PNGS knockout mutations at distal parts of Env rescued potent 1F7 neutralization of HIV-1 JR-CSF. Since the 1F7 epitope is disrupted on detergent-lysed virus Env extracts, it appears that knockout of PNGS at positions 156, 197, or 301 not only relieves quaternary restrictions but helps form a high-affinity binding site for 1F7 on the functional Env trimers. The removal of glycans at position 156, 197, or 301 [51] on JR-CSF gp120 also resulted in enhanced binding in our flow cytometry analysis using Env spikes displayed on the cell surface. The observed increase in neutralization sensitivity and trimer recognition by 1F7 induced by PNGS mutations could have been assisted at least in part by a repositioning of the V1/V2 loop [18], [75]. Notably, sensitivity to mAb VRC01 was considerably increased by knockout of the PNGS at position 197, whereas mutation of N301 had a minimal and N156 no effect at all. This is in line with the observations reported by Li et al., in which VRC01 was shown to be relatively indifferent to quaternary constraints [72]. Nevertheless, the presence of the PNGS at position 197, which is located on the base of V1/V2, is not *per se* sufficient to shield the CD4bs from access by 1F7, as illustrated by the fact that this PNGS is shared even by viruses that were found to be highly sensitive to 1F7 neutralization (Figure S3).

The importance of V1/V2 in the epitope of 1F7 was also borne out by experiments using chimeric isolates of JR-FL containing domains swapped with JR-CSF and *vice versa* (Figure 6). While the C3 region containing the CD4 binding loop surprisingly did not affect neutralization, a major impact was observed by swapping V1/V2 and C2 regions. The exact sequence and positioning of the V1/V2 loop in relation to residues in C2 therefore appear to modulate the 1F7 epitope. Interestingly, a JR-FL knock-in mutant carrying a glycan at position 197 was more resistant to neutralization than the wildtype virus JR-FL that lacks it, regardless of whether it was missing because of a mutation of N197 or S199. In contrast, binding to detergent-solubilized Env was influenced only by swaps with C2 and not by glycan. Thus, while VRC01 binds to an epitope involving residues of the V5 region, we hypothesize that the 1F7 epitope extends more towards the V1/V2 loop. Structural studies using 1F7 may in future be able to better define this hypothesis.

Overall, our results add to the growing evidence that the CD4bs, a highly conserved functional site on the Env trimer, can be targeted by many diverse and extremely potent and/or broadly neutralizing antibodies. Together with a previous study in which elicited antibodies were shown to compete with CD4bs antibodies and neutralized JR-CSF but not JR-FL [59], our data on 1F7 show how CD4bs epitopes on two related Env backgrounds are tightly controlled by sequence variation and proximal N-glycosylation on trimeric Env. During natural infection and often with immunization both monomeric and trimeric or other oligomeric states of gp120 can contribute to the humoral response to the CD4bs. However, both the quaternary occlusion effect, as well as the converse effect of the quaternary formation of epitopes around the CD4bs likely cause subtle shifts of proximal domains, glycans and variable loops. To properly understand how such shifts affect CD4bs immunogenicity will require high-resolution analysis of structures of trimeric Env and their associated B cell responses in relevant animal models.

## Conclusions

For vaccine design, it is logical to present the CD4bs in a form that includes the same binding constraints as imposed by the native trimer. However, because constraints on antibody binding to the CD4bs are isolate-specific, to favor broad VRC01-like antibodies, strategies must also account for CD4bs mAbs of the intermediate class to which 1F7, and the recently described HJ16 [41] belong. Thus, 1F7-type antibodies can at times bind even to native Env trimers of primary isolates (e.g. JR-FL) with high affinity, and, once elicited, could potentially outcompete broader, VRC01-like antibody responses. Probing the evolution of the host B cell response through approaches such as deep sequencing prior to and following vaccination may allow for tracking immunodominant specificities at the earliest stages. Empirical screens of immunogens in a variety of immunization regimens, alone or in polyvalent and/or prime-boost combinations will likely be necessary to reproducibly promote B cell lineages giving rise to CD4bs broadly neutralizing antibodies.

## Supporting Information

Figure S1 Neutralization activity of mAb 1F7 against a cross-clade panel of 157 pseudotyped viruses. Neutralization was performed in the Monogram Biosciences assay format. IC_50_ values for mAbs b12, 2G12, 2F5, 4E10, PG9, and PG16, which were obtained using the same assay, were taken from Walker and colleagues [35].
(TIFF)

Figure S2 MAb 1F7 does not bind to auto-antigens. Several mAbs were tested for reactivity against a panel of antigens (lipids, proteins, and dsDNA). MAb 4E10 served as positive control (open blue circles), 1F7 is depicted as red squares. Each assay was performed in duplicate. Data are representative of at least 3 repeats.
(TIFF)

Figure S3 Sequence alignment of 1F7-resistant and 1F7-sensitive gp120s. Only positions are shown that were considered relevant for b12 binding [51] as well as glycosylations at positions 156, 197, and 301. Color-coding: non-polar – yellow/brown; polar uncharged – pink/purple; polar negatively charged – light green/dark green; polar positively charged – light blue/black. IC_50_ values were derived from large-scale neutralization panel depicted in Figure S1.
(TIFF)
