# Supplementary figures and images for: A Human Antibody to the CD4 Binding Site of gp120 Capable of Highly Potent but Sporadic Cross Clade Neutralization of Primary HIV-1
Source: PLoS One. 2013 Aug 26;8(8):e72054. doi: 10.1371/journal.pone.0072054 (PMC3753353; doi:10.1371/journal.pone.0072054)

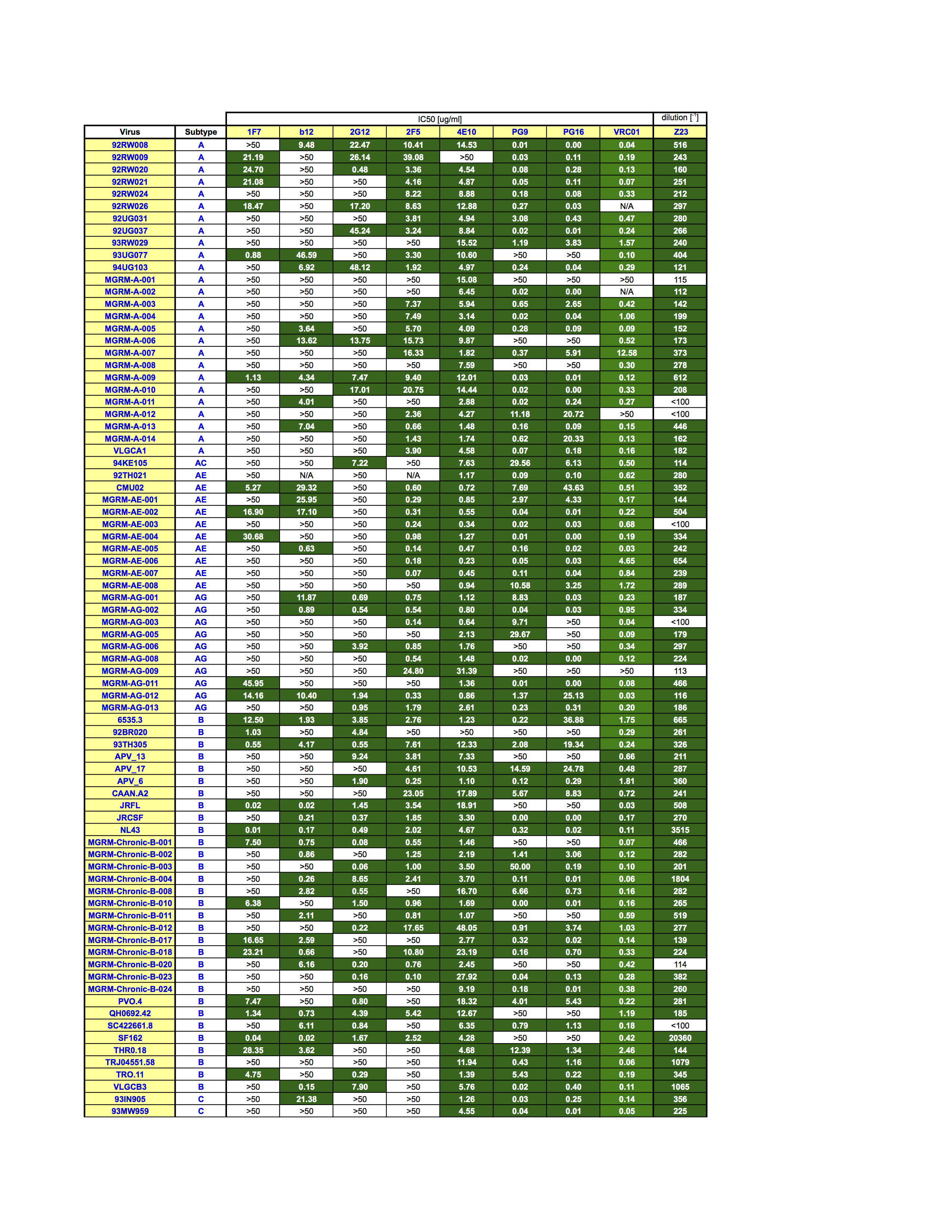

Supplement: Figure S1 — Neutralization activity of mAb 1F7 against a cross-clade panel of 157 pseudotyped viruses. Neutralization was performed in the Monogram Biosciences assay format. IC50 values for mAbs b12, 2G12, 2F5, 4E10, PG9, and PG16, which were obtained using the same assay, were taken from Walker and colleagues [35]. (TIFF) [file pone.0072054.s001.tiff]

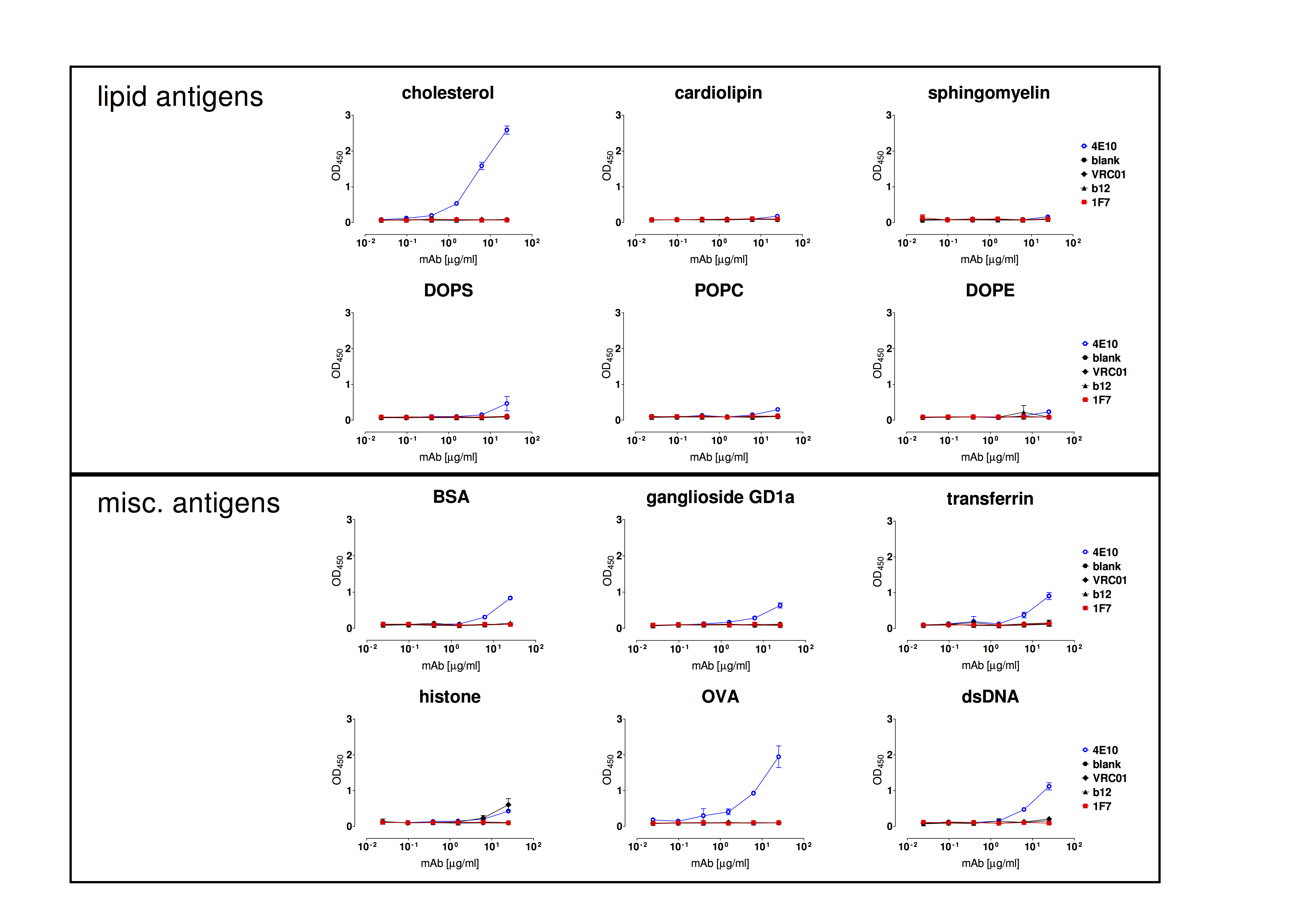

Supplement: Figure S2 — MAb 1F7 does not bind to auto-antigens. Several mAbs were tested for reactivity against a panel of antigens (lipids, proteins, and dsDNA). MAb 4E10 served as positive control (open blue circles), 1F7 is depicted as red squares. Each assay was performed in duplicate. Data are representative of at least 3 repeats. (TIFF) [file pone.0072054.s002.tiff]

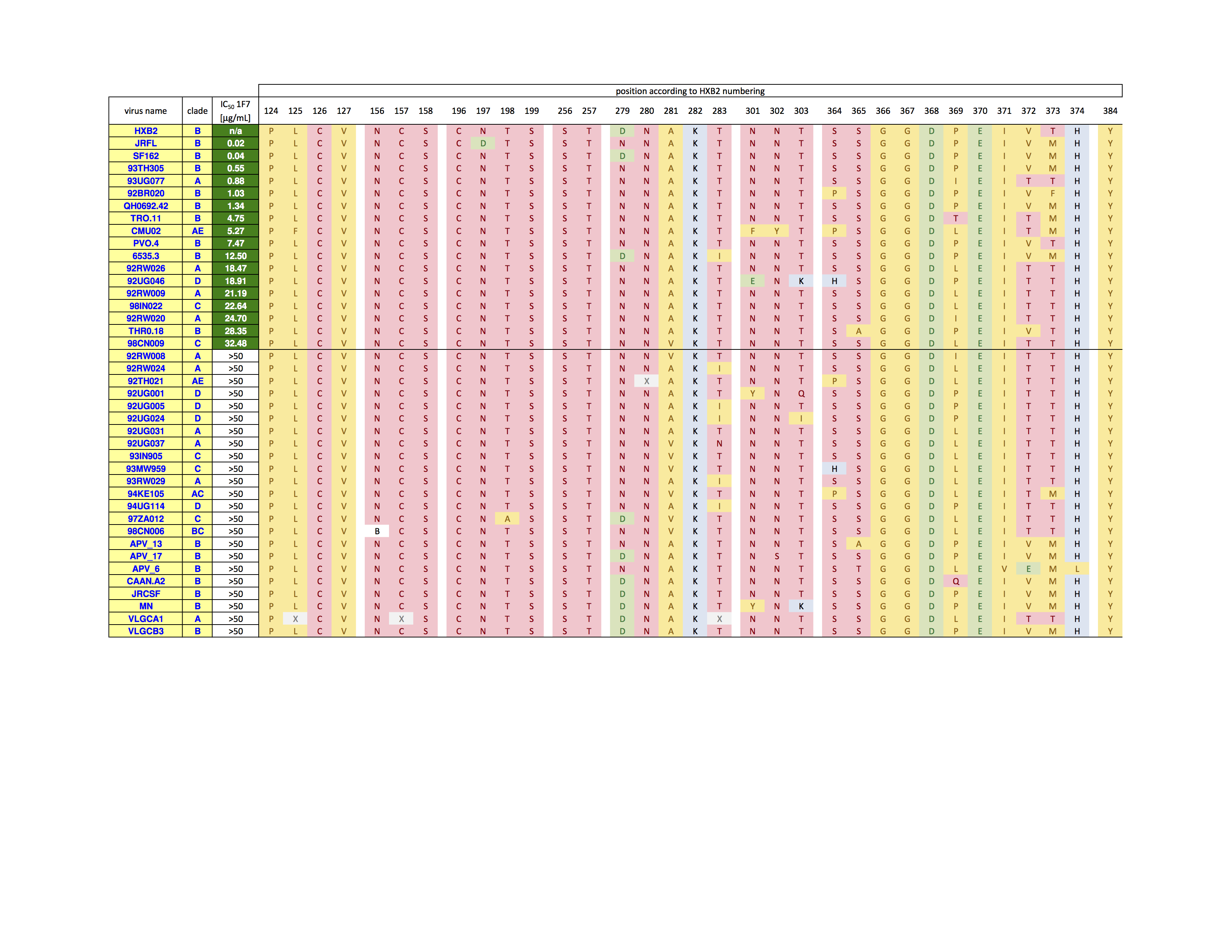

Supplement: Figure S3 — Sequence alignment of 1F7-resistant and 1F7-sensitive gp120s. Only positions are shown that were considered relevant for b12 binding [51] as well as glycosylations at positions 156, 197, and 301. Color-coding: non-polar – yellow/brown; polar uncharged – pink/purple; polar negatively charged – light green/dark green; polar positively charged – light blue/black. IC50 values were derived from large-scale neutralization panel depicted in Figure S1. (TIFF) [file pone.0072054.s003.tiff]
